# Generalized cutis marmorata telangiectatica congenita or neonatal lupus? A case report and literature review

**DOI:** 10.1002/ccr3.8898

**Published:** 2024-05-07

**Authors:** Shabnam Hajiani Ghotbabadi, Hamide Barzegar, Mitra Abdolvand

**Affiliations:** ^1^ Department of Rheumatology Shiraz University of Medical Sciences Shiraz Iran; ^2^ Neonatal Research Center Shiraz University of Medical Sciences Shiraz Iran; ^3^ Department of Dermatology, School of Medicine Molecular Dermatology Research Center, Shiraz University of Medical Science Shiraz Iran

**Keywords:** capillary telangiectasia, CMTC, cutis marmorata telangiectatic congenita, neonatal lupus syndrome, pediatrics

## Abstract

Cutis marmorata telangiectatica congenita (CMTC) presents as marbled erythema and may exhibit diverse associated anomalies. Thorough multidisciplinary evaluation is crucial. Treatment varies with inconclusive evidence, necessitating further research. Our case underscores CMTC's rarity and heterogeneous nature, advocating for comprehensive management approaches and ongoing research.

## 1 INTRODUCTION

Cutis marmorata telangiectatica congenita (CMTC) is an infrequent vascular anomaly present at birth.^1^, ^2^ It is characterized by persistent marbled erythema that pales under pressure.^1^ This slow‐flow vascular lesion, affecting capillaries and venules, may result in cutaneous atrophy, ulcerations, and potential associations with body asymmetry, as well as additional anomalies such as congenital glaucoma, limb asymmetry, and central nervous system involvement warranting medical attention.^3^, ^4^, ^5^ While the pathogenesis of CMTC remains unknown^6^ various genetic theories, such as autosomal mutation, mosaicism, and autosomal dominance have been reported in the literature.^7^ The diagnosis is primarily clinical.^4^, ^5^

In this case report we present an infant with generalized CMTC without any accompanying diseases or malformation and review the literature.

## 2 CASE HISTORY/EXAMINATION

We present the case of a male infant born at 37 +4 weeks gestational age from a 37 years old mother. The pregnancy was uneventful, with the only notable finding being a palpable lump in the left breast associated with stiffness during the last trimester. Ultrasonography revealed a large non‐mass area involving the 2–6 o'clock position of the left breast, characterized by increased echogenicity of fibrogranular tissue, mild soft tissue edema, and heightened vascularity. A biopsy was performed, confirming the diagnosis of granulomatous mastitis with micro abscess formation. Rheumatologic workup, including human leukocyte antigens (HLA)‐B5, HLA‐B51, anti‐cyclic citrullinated peptide, Rheumatoid Factor, anti Cardiolipin antibody, Antineutrophilic cytoplasmic antibody, anti‐double strand DNA, antinuclear antibody (ANA), Anti Beta2 glycoprotein IgG, and anti Beta2 glycoprotein IgM, yielded normal results. The mother also exhibited normal thyroid function.

**FIGURE 1:**
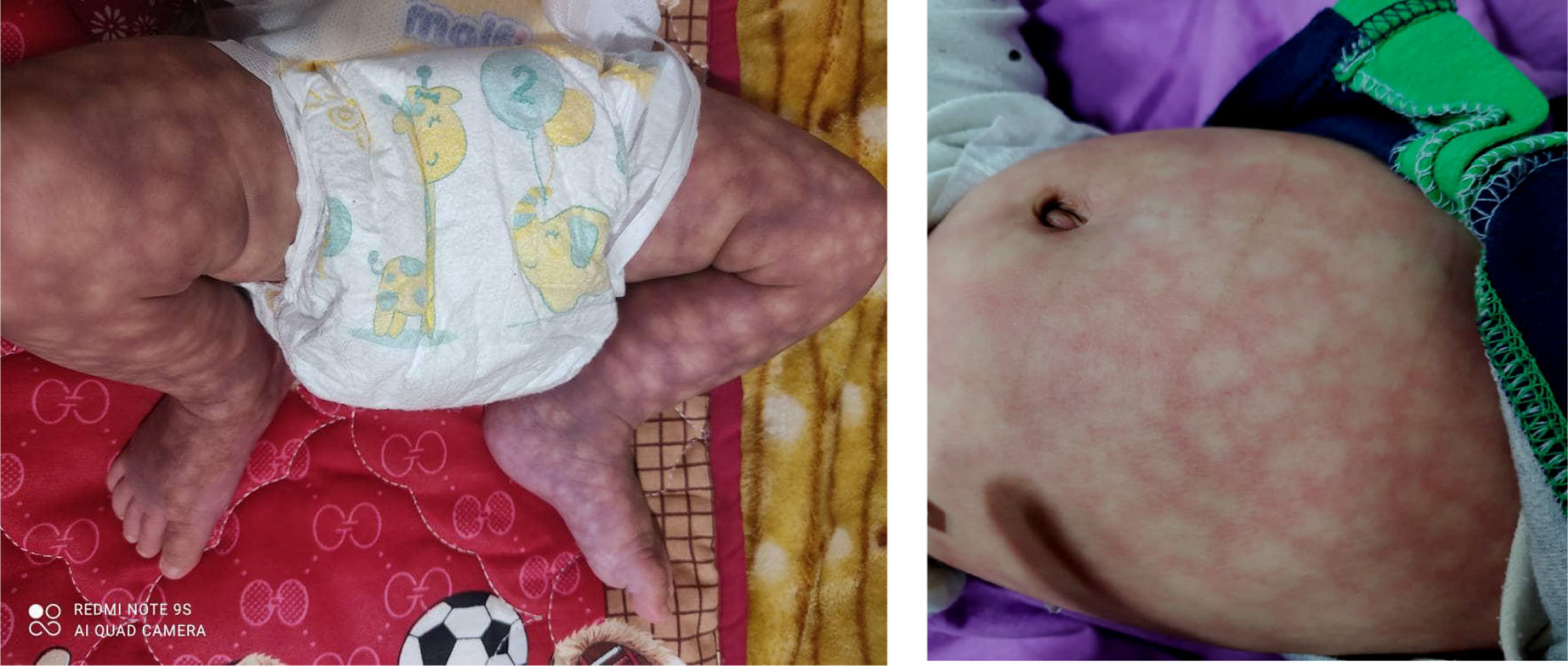
Net‐like pattern, marbled appearance generalized skin lesions.

The delivery was uneventful, the baby born with birth weight 2810 g (50 percentile), height 48 cm (25 percentile), and head circumference 33.5 cm (75 percentile). His APGAR scores were 8 and 10 at 1st and 5th minutes, respectively. The infant underwent a routine physical examination, revealing a mottled skin appearance resembling a net‐like pattern (Figure 1). No asymmetry was noted. Initial suspicions of hematologic and vascular disorders due to positive history of granulomatous mastitis in mother were dispelled, as the infant exhibited no bleeding tendency, and coagulation parameters were within normal limits.

The complete blood count revealed a white blood cell count of 6.12 × 103/μL, hemoglobin of 17.1 g/dL, and platelet count of 213 × 103/μL. Thyroid function (thyroid‐stimulating hormone: 4.6, T4: 13.4) was normal. Evaluation for neonatal lupus, including ANA, anti SS‐A, anti SS‐B, and anti Ds DNA, all fell within the normal range.

## 3 METHODS (DIFFERENTIAL DIAGNOSIS, INVESTIGATIONS AND TREATMENT)

**FIGURE 2:**
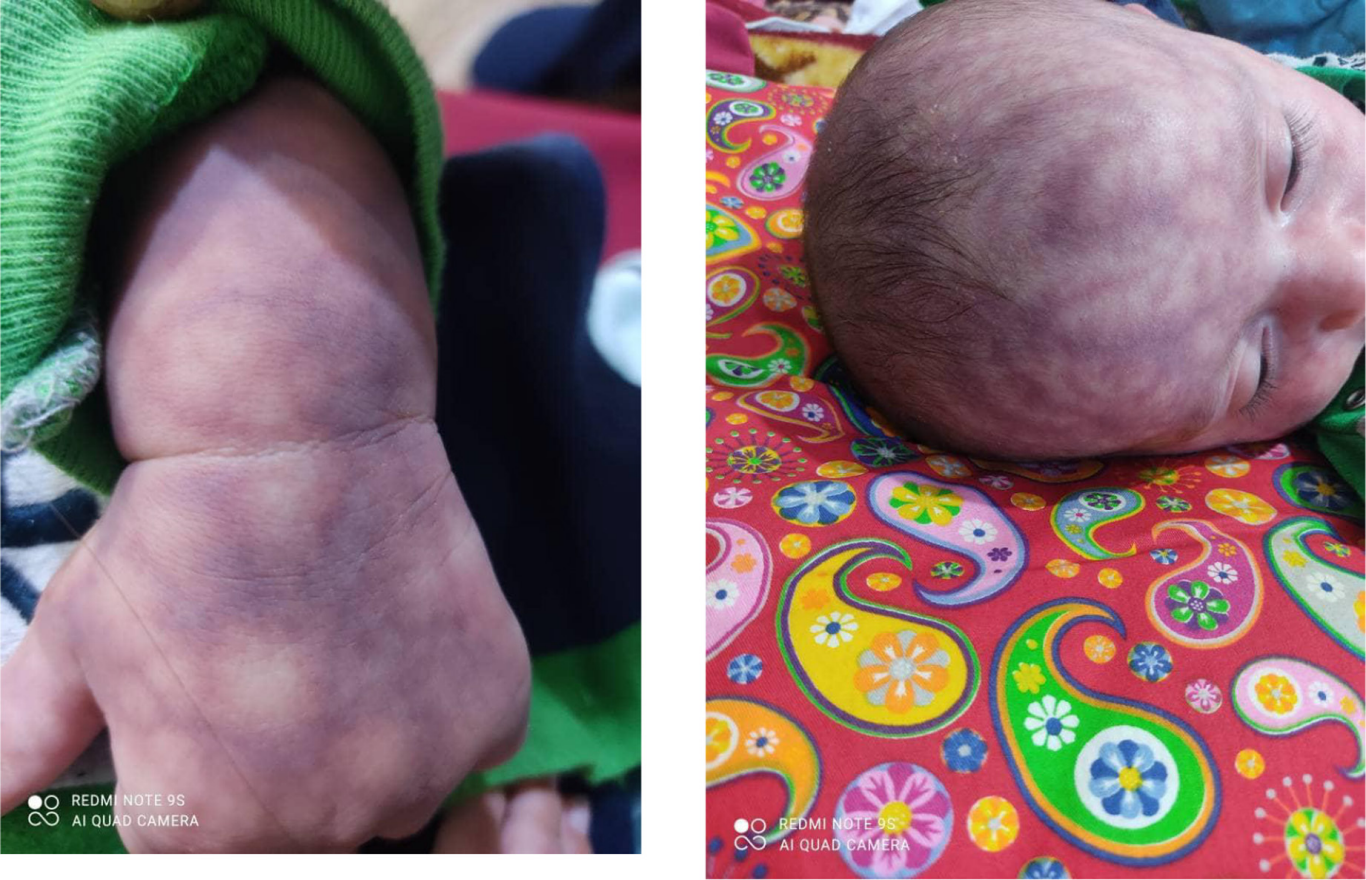
Skin lesions marbled pattern, exacerbated by fever, and lethargy.

At 3 months of age, the infant was admitted to the hospital due to poor feeding and fever. He presented with lethargy, a temperature of 38°C, pulse rate of 120/min, respiratory rate of 40/min, and blood pressure of 85/40. Skin examination revealed a mottled‐purple appearance exacerbated by fever (Figure 2). The patient's growth indices at 3 months included a height of 56 cm (50 percentile), head circumference of 40 cm (75 percentile), and weight of 6000 grams (75 percentile). He was referred to a pediatric rheumatologist to investigate vasculitis and neonatal lupus due to his mother's positive history of granulomatous mastitis. Then, he was hospitalized for a sepsis workup. Further investigations, including abdominopelvic sonography, brain sonography, and echocardiography revealed no abnormalities. Lumbar puncture showed no significant findings, and ophthalmologic examination yielded normal results. Additionally, rheumatologic tests were inconclusive, leading to the ruling out of neonatal lupus.

## 4 CONCLUSION AND RESULTS

Consequently, based on the provisional diagnosis of CMTC, dermatologist consultation was sought, confirming CMTC. After a four‐day hospital stay, ruling out sepsis as the cause of his symptoms, the infant was discharged in good condition.

## 5 DISCUSSION

Here, we present a case of an infant with generalized CMTC. While CMTC is a rare skin disorder that may or may not be accompanied by other anomalies, the generalized form is less common. In a study by Amitai et al.^8^ involving 85 patients with cutis marmorata, none exhibited the generalized form. However, Devillers et al.^9^ reported that 11% of 35 patients with CMTC had generalized involvement. Kienast et al.^1^ found only one case of generalized lesions out of 27 patients with CMTC. A recent literature review indicated that 24.5% of patients had generalized involvement.^2^ Overall, it seems that the localized form is more frequent.

Our presented case had no associated anomalies, though there are diverse reports of such associations in the literature. A literature review revealed that 42.5% of cases had associated anomalies.^2^ Other skin manifestations, including hemangioma, café‐au‐lait spots, pigmented nevus, and aplasia cutis, have been documented.^8^ Additionally, leg length discrepancy^5^ and glaucoma^2^ are reported as associated anomalies.

When CMTC is suspected, it is advisable to conduct a thorough evaluation with a multidisciplinary team. Treatment is usually not indicated, as these symptoms typically resolve within the first few years.^10^ Treatment for skin lesions in CMTC is varied, with reports indicating both effectiveness and ineffectiveness of laser therapy.^2^, ^11^, ^12^ Other interventions, such as brachial sympathectomy, have shown mixed results.^13^, ^14^ However, due to the limited number of studies, a standardized treatment strategy for skin lesions in CMTC cannot be recommended, highlighting the need for further research and clinical studies to establish more conclusive guidance.

Most mothers of infants with neonatal lupus are asymptomatic. Neonatal lupus, an autoimmune disease, occurs when antibodies cross the placenta to the fetus.^15^ The major primary presentations are cardiac and cutaneous manifestations. Typical rashes of neonatal lupus include erythematous annular lesions or arcuate macules with central atrophy.^15^, ^16^ Additionally, there are reports of neonatal lupus presenting as CMTC.^17^, ^18^

In conclusion, our presentation of an infant with generalized CMTC underscores the rarity of the generalized form and the variability in associated anomalies. Despite our case lacking associated anomalies, the literature reports a significant incidence of diverse anomalies in CMTC cases. The heterogeneous nature of CMTC, coupled with the absence of a standardized treatment strategy for skin lesions, highlights the need for comprehensive multidisciplinary evaluations when CMTC is suspected. Further research and clinical studies are imperative to guide the development of evidence‐based therapeutic approaches for this complex disorder, contributing to a more comprehensive understanding of CMTC and its optimal management.

## AUTHOR CONTRIBUTIONS

**Shabnam Hajiani Ghotbabadi:** Conceptualization; project administration; writing – original draft; writing – review and editing. **Hamide Barzegar:** Conceptualization; supervision; writing – original draft; writing – review and editing. **Mitra Abdolvand:** Data curation; writing – original draft; writing – review and editing.

## FUNDING INFORMATION

No funding was obtained for this study.

## CONFLICT OF INTEREST STATEMENT

The authors declare that they have no competing interests.

## ETHICS STATEMENT

The study protocol confirmed to the ethical guidelines of the 1975 Helsinki Declaration. The publication of this case was approved by the ethics committee of Shiraz University of Medical Sciences(IR.SUMS.MED.REC.1402.522). We have written informed consent obtained from the parents of the patient for publication of this case report.

## CONSENT

Written informed consent was obtained from the patient to publish this report in accordance with the journal's patient consent policy.

## Data Availability

All data generated or analyzed during this study are included in this published article.
